# The effects of supplementation with *P-Synephrine* alone and in combination with caffeine on resistance exercise performance

**DOI:** 10.1186/s12970-015-0096-5

**Published:** 2015-09-17

**Authors:** Nicholas A. Ratamess, Jill A. Bush, Jie Kang, William J. Kraemer, Sidney J. Stohs, Vincenzo G. Nocera, Megan D. Leise, Keith B. Diamond, Avery D. Faigenbaum

**Affiliations:** Department of Health and Exercise Science, The College of New Jersey, Ewing, NJ 08628 USA; Department of Human Sciences, The Ohio State University, Columbus, OH 43210 USA; School of Pharmacy and Health Professions, Creighton University, Omaha, NE 68178 USA

## Abstract

**Background:**

Little is known concerning the potential ergogenic effects of *p*-synephrine supplementation. Therefore, the purpose of the present study was to examine the effects of supplementation with *p*-synephrine alone and in combination with caffeine on free-weight resistance exercise performance.

**Methods:**

Twelve healthy, college-aged men performed a control (CT) resistance exercise protocol consisting of 6 sets of squats for up to 10 repetitions per set using 80 % of their one repetition-maximum (1RM) with 2 min of rest in between sets. Each subject was randomly assigned (in double-blind, balanced manner) to a treatment sequence consisting of use of 3 supplements: *p*-synephrine (S; 100 mg), *p*-synephrine + caffeine (SCF; 100 mg of *p*-synephrine plus 100 mg of caffeine), or a placebo (P). For each supplement treatment (separated by 1 week), subjects consumed the supplement for 3 days prior to each protocol and the morning of each protocol, and subsequently did not consume any supplements for 3 days following (i.e. wash-out period). On each protocol day, subjects reported to the lab at a standard time, consumed a supplement, sat quietly for 45 min, performed the resistance exercise protocol, and sat quietly for 30 min post exercise. Performance (repetition number, force, velocity and power), blood lactate, and ratings of perceived exertion (RPE) data were collected during each protocol.

**Results:**

Supplements SCF and S produced a significantly (*P* < 0.05) greater number of repetitions performed than CT (by 11.0 ± 8.0 %) and P (by 6.0 ± 7.0 %) and a 10.6 ± 12.0 % greater increase in volume load per protocol than CT and P. Most of the differences were seen during the last 3 sets. Mean power and velocity for all 6 sets were significantly higher in SCF compared to CT and P by ~6.2 ± 8.0 %. No supplement effects were observed in RPE or blood lactate, and no adverse side effects were observed or reported.

**Conclusions:**

S and SCF augmented resistance exercise performance (total repetitions, volume load) without increasing blood lactate or RPE. The addition of caffeine in SCF increased mean power and velocity of squat performance. These results indicate supplementation with S and SCF can enhance local muscle endurance during resistance exercise.

## Background

Thermogenic supplement use in various forms has increased in popularity among athletes and individuals targeting weight loss. These supplements target weight loss by increasing energy expenditure, the rate of fat oxidation, and by decreasing appetite [1–6]. Although their effects on anaerobic exercise performance have been equivocal [7–10], thermogenic supplements are thought to have mild ergogenic properties. Many thermogenic supplements contain compounds such as *p*-synephrine and caffeine [11]. *p*-Synephrine is the primary protoalkaloid derived from the immature fruits of *Citrus aurantium* (bitter orange, Seville orange). *p*-Synephrine also occurs in other orange-related species as Marrs sweet oranges, clementines and mandarin oranges [12, 13].

*p*-Synephrine has its hydroxyl group located in the para position on its benzene ring which has been shown to significantly alter its adrenergic receptor binding properties. It has low binding affinity for α-1 and α-2 as well as β-1 and β-2 adrenoreceptors [14]. Therefore, it exhibits little or no cardiovascular stimulation compared to ephedrine, *m*-synephrine (phenylephrine), and the catecholamines [14]. The ergogenic actions of *p*-synephrine are thought to be mediated mostly by β-3 adrenoreceptor activation leading to increased metabolic rate, lipolysis, and possibly reduced food intake [14, 15].

Stohs et al. [12] reviewed over 20 published and unpublished human studies involving *p*-synephrine with doses ranging from 5 to 80 mg (alone and in combination with 132 to 704 mg of caffeine). *p*-Synephrine alone or in combination with other nutrients increased resting metabolic rate and energy expenditure by up to ~13 %, and led to modest reductions in body weight (all of the studies examining weight loss investigated *p*-synephrine in combination with other nutrients). However, potential ergogenic effects of *p*-synephrine are poorly understood particularly during resistance exercise.

Caffeine is a thermogenic methylxanthine alkaloid that acts as a central nervous system stimulant. It has been shown to increase energy expenditure and lipolysis and fat oxidation [16]. Caffeine is thought to enhance performance via several mechanisms including adenosine antagonism, potentiated cyclic AMP via phosphodiesterase inhibition, augmented muscle glycogen resynthesis, increased β-endorphin and catecholamine secretion, increased nerve conduction velocity and motor unit recruitment, reduced pain perception, and enhanced permeability, mobilization, and reduced uptake of intracellular calcium [16–22]. Some studies have shown caffeine consumption of 3–6 mg/kg of body mass increases endurance performance [16]. However, caffeine consumption during resistance exercise has produced equivocal findings. Some studies have shown ergogenic effects on muscle strength and endurance performance [23, 24], while others have failed to show performance augmentation [25, 26]. A meta-analysis has shown that caffeine consumption increases muscle strength and endurance but the effects may be muscle-group specific [27].

Given the paucity of studies examining *p*-synephrine supplement consumption during resistance exercise, the purpose of the present study was to examine resistance exercise performance concomitant with consumption of *p*-synephrine with or without a low dose of caffeine. It was our hypothesis that *p*-synephrine with and without caffeine would enhance repetition and power performance during resistance exercise.

## Methods

### Experimental design

In order to examine the primary hypotheses of the present study, a double-blinded, randomized, within-group crossover study design was used. After familiarization and preliminary baseline resistance exercise performance testing, subjects were randomly assigned (using a balanced design) to either a *p*-synephrine (100 mg; S), *p*-synephrine + caffeine (100 mg *p*-synephrine plus 100 mg caffeine; SCF), or placebo (P) treatment. The resistance exercise protocol consisted of performing 6 sets of back squats for up to 10 repetitions per set using 80 % of subjects’ maximal strength with 2 min of rest in between sets at a standard time early in the morning in a fasted state. The protocol was performed 4 times (baseline and following consumption of each supplement). Subjects consumed each supplement in the form of chews for 3 days prior to and upon arrival at the laboratory the day of each protocol and then washed-out for 3 days following each protocol day. The 3-day pre-protocol supplementation period was utilized in order to expose subjects to the supplements on multiple occasions (to examine how subjects tolerated the supplemental doses) prior to performance assessment. Performance (repetition number, force, velocity, and power), blood lactate, and ratings of perceived exertion data were collected during each protocol.

### Subjects

The subjects were healthy, college-aged (e.g., 20–26 years) men (*N* = 12) who were former athletes with at least 2 years of resistance training experience from the student population at The College of New Jersey. Descriptive subject characteristics are shown in Table 1. None of the subjects were taking any medications, anabolic steroids, or nutritional supplements known to affect resistance exercise performance. All subjects had refrained from caffeine intake for at least 3 weeks prior to starting the study. Each subject was monitored during this time for symptoms such as headaches and fatigue in order to minimize potential confounding effects of caffeine withdrawal reversal. Three weeks was selected as withdrawal effects from low-to-moderate caffeine consumption typically subside within 10 days [28]. No withdrawal effects were reported upon initiation of pre-study familiarization and testing. Prior to this period, 5 subjects reported low daily caffeine intake (<10–20 mg/day), 3 subjects reported moderate caffeine intake (100–200 mg/day), and 4 subjects reported high daily caffeine intake (250–400 mg/day). Subjects were consistently questioned, completed diet records, and instructed not to consume known sources of caffeine during the experimental period. None of the subjects had any physiological or orthopedic limitations that could have affected lifting performance as determined by completion of a health history questionnaire prior to initiating the study. Subjects were instructed to refrain from exercise for 2 days prior to each protocol. This study was approved by The College of New Jersey’s Institutional Review Board and each subject subsequently signed an informed consent document prior to participation.

**Table 1:** Subject descriptive characteristics

Variable	Mean ± *SD*
Age (yrs)	22.3 ± 1.6
Height (cm)	179.5 ± 7.8
Body mass (kg)	81.3 ± 9.2
RT experience (yrs)	5.8 ± 2.4
Percent body Fat (%)	10.7 ± 3.9
VO_2_ max (ml ⋅ kg^−1^ ⋅ min^−1^)	50.3 ± 7.2
1RM squat (kg)	135.6 ± 24.4
80 % of 1RM squat (kg)	107.2 ± 19.2

Key: *RT* Resistance training, *VO*_*2*_ Oxygen consumption, *1RM* One repetition-maximum

### Preliminary screening and familiarization

During the first visit to the Human Performance Laboratory, subjects completed a medical history questionnaire, informed consent document, were familiarized with the equipment and procedures, and were instructed on how to properly complete a 3-day dietary record. Height was measured using a wall-mounted stadiometer and body mass was measured using an electronic scale. VO_2_ max was assessed using a progressive, multi-stage ramp protocol on a treadmill using a metabolic system (MedGraphics ULTIMA Metabolic System, MedGraphics Corporation, St. Paul, MN). Percent body fat was estimated via a three-site skinfold test. The sites measured were the pectoral, anterior thigh, and abdominal skinfolds [29]. The same research assistant performed all skinfold assessments. Body density was calculated using the equation of Jackson and Pollock [29] and percent body fat was calculated using the equation of Siri [30]. Dietary records were used to ensure subjects maintained normal kilocalorie and macronutrient intake throughout the study, and were completed for 3 days prior to the baseline protocol and for 3 days prior to each subsequent protocol. Each dietary record was analyzed using the Nutrition Calc Plus Version 3.4 software program (ESHA Research, Salem, OR). Total kilocalories, grams of fat, carbohydrates, and protein, and percent dietary intake of fats, carbohydrates, and protein were analyzed.

### Strength testing

The one-repetition maximum (1RM) squat was used as a measure of strength using a standard protocol [31]. A warm-up set of 5–10 repetitions was performed using 40–60 % of the perceived 1RM. After a 1-min rest interval, a set of 2–3 repetitions was performed at 60–80 % of the perceived 1RM. Subsequently, 2–4 maximal trials were performed to determine the 1RM with 2–3 min rest intervals in between trials. A complete range of motion and proper technique was required for each successful 1RM trial. Subjects descended with the bar on the rear shoulders until their upper thighs were parallel to the ground. At that point a “lift” signal was given by a research assistant (to ensure proper depth) and the subject ascended to the starting position. Assessment of 1RM strength enabled calculation of the protocol loads (i.e. 80 % of 1RM).

### Control protocol

A control (no supplement) resistance exercise protocol was performed first to establish baseline resistance exercise performance. Subjects reported to the laboratory early in the morning following a 10-h fast to minimize any confounding influence from prior meal consumption. Only water consumption was permitted. Upon arrival, each subject was encouraged to drink water *ad libitum* to pre-hydrate. Subjects had a cannula inserted into an antecubital vein for blood sampling. Subsequently, each subject was positioned on a reclining chair and sat quietly for 15 min prior to measurement of baseline blood lactate. Subjects then proceeded to sit quietly for an additional 45 min in a recumbent position. The quiet sitting period was used to mimic the time of supplement consumption during subsequent protocol sessions.

Subjects performed a standard warm-up consisting of 3 min of stationary cycling or walking, light stretching, and 2–3 light sets of squats with 30–65 % of 1RM. Water was provided *ad libitum* during this time. The resistance exercise protocol consisted of performing 6 sets of up to 10 repetitions (i.e. when momentary muscular failure was attained prior to the completion of 10 repetitions) of the free-weight back squat with ~80 % of 1RM using 2-min rest intervals in between sets. Standard exercise technique was used and only those repetitions that met the criteria were counted. Resistance remained constant while total numbers of repetitions were recorded. Subjects used a self-selected cadence (with no rest in between repetitions) in order to maximize resistance exercise performance. Following each set, ratings of perceived exertion (RPE) were obtained using a category ratio (CR) 10-point (0–10) scale. Volume load (kg) was calculated as the number of completed repetitions x resistance used.

### Experimental protocol

Following the control session, subjects were randomly assigned (in double-blind manner) to a balanced treatment sequence. The treatments involved use of 3 supplements: *p*-synephrine (100 mg), *p*-synephrine + caffeine (100 mg of *p*-synephrine plus 100 mg of caffeine), or a placebo treatment. Subjects consumed random treatment 1 for 3 consecutive days prior to returning to the laboratory. Subjects then reported to the laboratory the following morning in a fasted state similar to the control protocol session. After baseline measures and blood sampling, subjects consumed a dose of treatment 1. They sat quietly for 45 min and subsequently initiated the resistance exercise protocol discussed previously. Each subject did not consume a supplement for the next 3 days (i.e. a 3-day “wash-out” period was used based on the half-lives and patterns of elimination of *p*-synephrine and caffeine) but then began supplementing with treatment 2 for 3 days prior to their next scheduled protocol session. Subjects arrived at the laboratory, consumed treatment 2, sat for 45 min, and repeated the protocol described above. Following a 3-day wash-out period, subjects consumed treatment 3 for 3 days and repeated the protocol in a similar manner. Each supplement protocol session was identical to the control session with the exception of supplement consumption prior to the 45 min quiet sitting period (following baseline assessments). This cross-over design allowed each subject to experience all supplemental conditions in random sequence.

### Resistance exercise kinetics and kinematics

Each resistance exercise protocol was performed on a portable force plate (Advanced Medical Technology Inc., Watertown, MA) with data collected at a frequency of 200 Hz. Average and peak concentric ground reaction force per repetition were recorded and analyzed. Average bar velocity and power for the each repetition was measured with a Tendo™ Power Output Unit (Tendo Sports Machines, Trencin, Slovak Republic). The Tendo™ unit consists of a linear position transducer attached to the end of the barbell which measured linear displacement and time. Subsequently, average bar velocity and power were determined for each repetition. Power and velocity were averaged for each set (for all completed repetitions) and for each protocol. Test-retest reliability for the Tendo™ unit in our laboratory has consistently shown *R* > 0.90 [32].

### Blood lactate measurements

Subjects arrived at the laboratory in the early morning (at a standard time of day for all sessions) following an overnight fast. Venous blood samples were collected from subjects in a seated, semi-recumbent position at rest (T1), following the 45 min quiet sitting protocol (T2), immediately post-exercise (T3), and 15 min (T4), and 30 min (T5) post exercise. All blood samples were obtained using a 20-gauge Teflon cannula placed in a superficial forearm vein. The cannula was maintained patent via infusion of a heparin solution and blood was removed via a plastic syringe connected to a 3-way stopcock with a male luer lock adapter. T1 blood samples were drawn following a 15 min equilibration period. T3 blood samples were taken within 30 s of exercise cessation. Blood samples were collected into a Vacutainer® tube containing SST® Gel and Clot Activator. An aliquot of each whole blood sample was removed and immediately used for determination of blood lactate. Whole blood lactate was analyzed in duplicate using an Analox GM7 enzymatic metabolite analyzer (Analox Instruments USA, Lunenburg, MA).

### Supplement composition and procedures

The supplements used in the present study were in “chew” form (*Advantra Z®,* Nutratech, Inc., West Caldwell, NJ). Each chew was identical in appearance, chocolate flavored, and identical in taste. All three supplements contained isomalt, maltitol syrup, cocoa powder, natural flavors, palm oil, soy lecithin, glycerin, and stevia. One serving consisted of two chews. Each serving of chews contained 30 kcal from 8 g of carbohydrates. Thus, the placebo contained only these nutrients. The chews contained these nutrients with 100 mg of *p*-synephrine from *Citrus aurantium* extract in two chews. The *p*-synephrine + caffeine chews consisted of the same nutrients plus 100 mg of *p*-synephrine plus 100 mg of caffeine. The supplements were administered to subjects in absolute doses. The rationale was to examine the supplement in a manner consistent with manufacturer’s recommendations and to expand the *p*-synephrine literature base by providing a larger amount compared to previous studies which have mostly investigated absolute doses of 5–80 mg per day [12].

Subjects were instructed to consume each supplement for 3 days prior to arriving to the Human Performance Laboratory. Subjects were given the chews (6 in total) in bags labeled “A”, “B”, or “C”, specific instructions on consumption, forms to complete by recording the time of day each supplement was consumed, and were instructed to return the empty wrappers as documentation of use. Two chews were consumed each day generally during the late morning, early afternoon hours. Both chews were consumed simultaneously and subjects were instructed to chew them completely and hold remnants under the tongue for at least 2 min prior to swallowing. Subjects were also given two chews during each resistance exercise protocol, i.e. prior to the 45-min quiet sitting period resulting in consumption of 8 chews in total per treatment condition.

### Statistical analyses

Descriptive statistics (means ± *SD*) were calculated for all dependent variables. A 2-way (treatment x time point) analysis of variance (ANOVA) with repeated measures was used to analyze all within-subject data. Subsequent Tukey’s *post hoc* tests were utilized to determine differences when significant main effects were obtained. A one-way ANOVA was used to analyze diet records and body weight changes. Pearson-product moment correlations were calculated for selected variables. For all statistical tests, a probability level of *P* ≤ 0.05 denoted statistical significance.

## Results

### Dietary intake, body weight, and side effects

Statistical analysis of the 3-day dietary records showed that subject nutritional intake did not vary between the four experimental conditions. Subjects consumed an average of 2,594.2 ± 568.3 kcals per day. Daily intake of protein, carbohydrates, and fat averaged 159.3 ± 53.8, 279.4 ± 96.7 and 95.3 ± 27.8 g, respectively. This equated to 24.6 ± 6.9, 42.4 ± 5.9 and 32.3 ± 5.4 % of daily kilocaloric intake, respectively. Body mass did not significantly differ during each experimental protocol; CT = 82.0 ± 9.2 kg; P = 81.7 ± 9.4 kg; S = 82.0 ± 9.8 kg; and SCF = 81.6 ± 9.4 kg. Subjects reported no difference in taste between supplements as the chocolate flavor masked any potential bitter taste of the nutrients. In addition, no adverse side effects were reported throughout the experimental period.

### Repetition performance and volume load

Table 2 presents repetition performance data. No supplement effect was observed when analyzing repetition number per set. However, significant differences were observed [*P* = 0.05] between treatments when total repetitions were analyzed. Supplements SCF and S produced significantly greater numbers of repetitions performed than CT and P. SCF and S produced ~11.0 ± 8.0 % greater repetition numbers than the CT condition and ~6.0 ± 7.0 % greater repetition numbers than the P treatment. In addition, a trend (*P* = 0.06) was observed where most of the differences shown between supplement conditions occurred between sets 4 and 6. A significant difference was observed [*P* = 0.03] between treatments. Supplements SCF (4,609.1 ± 1,175.2 kg) and S (4,623.0 ± 1,117.5 kg) produced a significantly greater volume load per protocol than CT (4,128.0 ± 1,258.1 kg) and P (4,321.6 ± 1,136 kg). A significant main effect was observed [*P* < 0.0001] where repetition performance decreased with each successive set during all treatments. Specific differences are shown in Table 2.

**Table 2:** Repetition performance data

	CT	P	S	SCF
Set 1	10.0 ± 0.0	10.0 ± 0.0	10.0 ± 0.0	10.0 ± 0.0
Set 2	8.1 ± 2.3^a^	9.2 ± 1.1	9.3 ± 1.2	8.9 ± 1.7
Set 3	6.6 ± 2.5^ab^	6.7 ± 3.0^ab^	7.8 ± 3.2^ab^	7.7 ± 2.3^ab^
Set 4	6.0 ± 2.7^abc^	5.9 ± 3.1^ab^	6.8 ± 3.1^ab^	6.5 ± 2.8^abc^
Set 5	4.8 ± 3.0^abcd^	5.4 ± 3.4^abc^	5.4 ± 2.8^abcd^	5.7 ± 3.1^abcd^
Set 6	4.0 ± 2.7^abcd^	4.1 ± 2.5^abcde^	4.9 ± 3.0^abcd^	5.3 ± 3.4^abcd^
Reps sets 1 – 3	24.7 ± 4.3	25.9 ± 3.6	27.1 ± 3.8	26.6 ± 3.7
Reps sets 4 – 6	14.8 ± 8.1	15.4 ± 8.7	17.1 ± 8.5^#^	17.5 ± 9.2^#^
Total reps	39.5 ± 12.1	41.3 ± 11.9	44.2 ± 11.7*	44.1 ± 12.4*

Values are mean ± SD

Key: *CT* Control protocol, *P* Placebo treatment, *S p*-synephrine treatment, *SCF p*-synephrine plus caffeine treatment

^a^ – *P* ≤ 0.05 from set 1; ^b^ – *P* ≤ 0.05 from set 2; ^c^ – *P* ≤ 0.05 from set 3; ^d^ – *P* ≤ 0.05 from set 4; ^e^ – *P* ≤ 0.05 from set 5; * - *P* ≤ 0.05 from CT and P; ^#^ - *P* = 0.06 from CT and P

### Ratings of Perceived Exertion (RPE)

Table 3 presents RPE data following each set of resistance exercise. No treatment effects were observed over all 6 sets and no significant treatments effects were observed for the mean session RPE [*P* = 0.18]. RPE obtained during each set was significantly [*P* < 0.001] negatively correlated with repetitions completed (*r* = −0.48 to −0.67). A significant time effect was observed between the 1st and 6th set of resistance exercise [*P* < 0.0001] where RPE values increased with each successive set.

**Table 3:** Ratings of perceived exertion

	CT	P	S	SCF
Set 1	5.36 ± 1.5	5.18 ± 1.60	5.14 ± 1.27	5.41 ± 1.46
Set 2	6.55 ± 1.6^a^	6.55 ± 2.02^a^	6.23 ± 1.57^a^	6.36 ± 1.63^a^
Set 3	7.14 ± 1.10^ab^	7.05 ± 1.98^ab^	6.73 ± 1.79^ab^	7.00 ± 1.84^ab^
Set 4	7.55 ± 1.29^abc^	7.59 ± 1.71^abc^	7.18 ± 1.94^abc^	7.50 ± 1.72^abc^
Set 5	7.91 ± 1.22^abc^	7.91 ± 1.51^abc^	7.50 ± 1.66^abc^	7.73 ± 1.68^abc^
Set 6	8.91 ± 1.22^abcde^	8.55 ± 1.37^abcde^	8.27 ± 1.49^abcde^	8.36 ± 1.36^abcde^
Mean	7.23 ± 1.12	7.14 ± 1.58	6.84 ± 1.49	7.06 ± 1.44

Values are mean ± SD

Key: *CT* Control protocol, *P* Placebo treatment, *S p*-synephrine treatment, *SCF p*-synephrine plus caffeine treatment

^a^ – *P* ≤ 0.05 from set 1; ^b^ – *P* ≤ 0.05 from set 2; ^c^ – *P* ≤ 0.05 from set 3; ^d^ – *P* ≤ 0.05 from set 4; ^e^ – *P* ≤ 0.05 from set 5

### Average and peak power

Average power data are presented in Table 4. No supplement effect was observed with regard to the pattern of reduction during each set. The mean power for all 6 sets was significantly higher in SCF compared to CT and P by ~6.2 ± 8.0 % [*P* = 0.03]. Although mean power during S was higher than CT and P, this value did not reach statistical significance. Average power was significantly negatively correlated to blood lactate values at T3 (*r* = −0.29 to −0.48). A significant time effect was observed [*P* < 0.0001] where average power decreased with each successive set. No supplement effects were observed with regard to the pattern of peak power reduction during each set. No significant differences were observed [*P* = 0.67] between treatments when examining the mean peak powers of all 6 sets (CT = 1315.2 ± 195.9 W; P = 1289.8 ± 170.6 W; S = 1294.0 ± 174.4 W; SCF = 1329.1 ± 197.2 W). A significant time effect was observed [*P* < 0.0001] where peak powers decreased with each successive set (data not shown).

**Table 4:** Average power data (Watts)

	CT	P	S	SCF
Set 1	827.7 ± 160.8	870.4 ± 153.9	860.8 ± 155.0	875.6 ± 160.4
Set 2	734.3 ± 127.2^a^	756.7 ± 144.3^a^	777.5 ± 160.9^a^	794.4 ± 152.5^a^
Set 3	681.2 ± 126.6^ab^	677.1 ± 119.2^ab^	703.6 ± 186.4^ab^	729.7 ± 150.5^ab^
Set 4	653.1 ± 134.1^ab^	622.6 ± 133.7^abc^	642.8 ± 169.4^abc^	682.4 ± 144.6^abc^
Set 5	600.9 ± 113.8^abcd^	598.2 ± 131.2^abc^	622.8 ± 140.2^abc^	644.7 ± 153.0^abcd^
Set 6	595.1 ± 100.5^abcd^	574.4 ± 114.6^abcde^	561.4 ± 121.4^abcde^	614.8 ± 141.8^abcde^
Mean	697 ± 108.6	697 ± 106.5	718.8 ± 124.6	743.0 ± 129.3*

Values are mean ± SD

Key: *CT* Control protocol, *P* Placebo treatment, *S p*-synephrine treatment, *SCF p*-synephrine plus caffeine treatment

^a^ – *P* ≤ 0.05 from set 1; ^b^ – *P* ≤ 0.05 from set 2; ^c^ – *P* ≤ 0.05 from set 3; ^d^ – *P* ≤ 0.05 from set 4; ^e^ – *P* ≤ 0.05 from set 5; * - *P* ≤ 0.05 compared to CT and P

### Average and peak velocity (m/s)

Average velocity data are presented in Table 5. No supplement effect was observed with regard to the pattern of reduction during each set. The mean velocity per set was significantly greater [*P* = 0.04] in SCF than CT and P by ~6.5 ± 8.0 %. Although mean velocity during S was higher than CT and P, this value did not reach statistical significance. A significant time effect was observed [*P* < 0.0001] where average velocity decreased with each successive set. No supplement effect was observed with regard to the pattern of peak velocity reduction during each set. No significant differences were observed [*P* = 0.84] between treatments when examining the mean peak velocities of all 6 sets (CT = 0.71 ± 0.06 m⋅s^−1^; P = 0.70 ± 0.10 m⋅s^−1^; S = 0.70 ± 0.07 m⋅s^−1^; SCF = 0.72 ± 0.08 m⋅s^−1^). A significant time effect was observed [*P* < 0.0001] where peak velocities decreased with each successive set (data not shown).

**Table 5:** Average velocity data (m ⋅ s^−1^)

	CT	P	S	SCF
Set 1	0.46 ± 0.06	0.49 ± 0.06	0.47 ± 0.06	0.48 ± 0.05
Set 2	0.41 ± 0.05^a^	0.42 ± 0.07^a^	0.43 ± 0.07^a^	0.44 ± 0.07^a^
Set 3	0.38 ± 0.06^ab^	0.38 ± 0.07^ab^	0.38 ± 0.09^ab^	0.40 ± 0.07^ab^
Set 4	0.36 ± 0.06^ab^	0.34 ± 0.07^abc^	0.35 ± 0.09^abc^	0.38 ± 0.08^abc^
Set 5	0.33 ± 0.06^abcd^	0.33 ± 0.08^abc^	0.34 ± 0.07^abc^	0.35 ± 0.08^abcd^
Set 6	0.33 ± 0.06^abcd^	0.32 ± 0.07^abcde^	0.31 ± 0.07^abcde^	0.34 ± 0.08^abcde^
Mean	0.38 ± 0.05	0.38 ± 0.06	0.39 ± 0.07	0.40 ± 0.07*

Values are mean ± SD

Key: *CT* Control protocol, *P* Placebo treatment, *S p*-synephrine treatment, *SCF p*-synephrine plus caffeine treatment

^a^ – *P* ≤ 0.05 from set 1; ^b^ – *P* ≤ 0.05 from set 2; ^c^ – *P* ≤ 0.05 from set 3; ^d^ – *P* ≤ 0.05 from set 4; ^e^ – *P* ≤ 0.05 from set 5; * - *P* ≤ 0.05 compared to CT and P

### Peak and average concentric force (N)

Peak concentric force data are presented in Table 6. No supplement effect was observed with regard to the pattern of peak force reduction during each set. No significant differences were observed [*P* = 0.21] between treatments when examining the mean peak concentric force of all 6 sets. Although S and SCF yielded higher peak concentric force (up to ~2.5 %) than CT and P, respectively, these differences did not reach statistical significance. A significant time effect was observed [*P* < 0.0001] where peak concentric force decreased with each successive set. No supplement effect was observed with regard to the pattern of average force reduction during each set. The mean concentric force across all 6 sets did not significantly differ among conditions (CT = 1875.9 ± 229.6 N; P = 1901.1 ± 210.5 N; S = 1898.1 ± 217.0 N; SCF = 1902.7 ± 222.9 N) [*P* = 0.24]. A significant time effect was observed [*P* = 0.03] where average concentric force decreased from set 1 through set 6 at various points (data not shown).

**Table 6:** Peak concentric force data (N)

	CT	P	S	SCF
Set 1	2256.5 ± 290.3	2295.1 ± 281.6	2287.8 ± 286.2	2328.8 ± 306.5
Set 2	2232.6 ± 276.9	2262.0 ± 296.4^a^	2265.9 ± 283.2	2301.0 ± 289.7
Set 3	2217.4 ± 249.5	2249.4 ± 272.0^a^	2252.7 ± 259.0^a^	2277.1 ± 283.6^ab^
Set 4	2207.0 ± 254.8^a^	2224.5 ± 270.8^a^	2251.9 ± 280.7^a^	2262.0 ± 287.0^ab^
Set 5	2184.1 ± 258.4^ab^	2208.4 ± 251.9^abc^	2224.9 ± 267.8^ab^	2227.5 ± 268.6^abcd^
Set 6	2175.3 ± 252.9^ab^	2170.9 ± 240.4^abcde^	2209.7 ± 244.0^abc^	2214.3 ± 241.6^abcd^
Mean	2212.1 ± 258.0	2235.0 ± 266.7	2248.8 ± 266.9	2268.5 ± 277.1

Values are mean ± SD

Key: *CT* Control protocol, *P* Placebo treatment, *S p*-synephrine treatment, *SCF p*-synephrine plus caffeine treatment

^a^ – *P* ≤ 0.05 from set 1; ^b^ – *P* ≤ 0.05 from set 2; ^c^ – *P* ≤ 0.05 from set 3; ^d^ – *P* ≤ 0.05 from set 4; ^e^ – *P* ≤ 0.05 from set 5

### Blood lactate

**Fig. 1:**
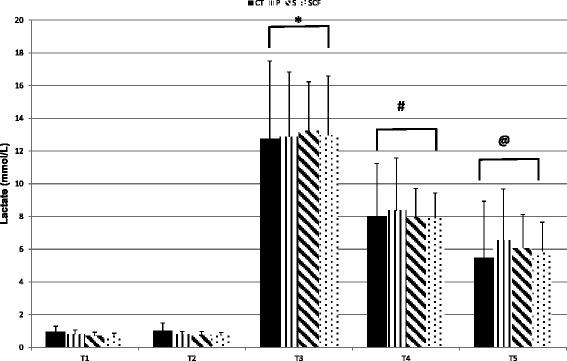
Blood Lactate Response. Key: *CT* Control protocol, *P* Placebo treatment, *S p*-synephrine treatment, *SCF p*-synephrine plus caffeine treatment; * – *P* ≤ 0.05 from T1, T2, T4, and T5; # – *P* ≤ 0.05 from T1, T2, T3, and T5; @ – *P* ≤ 0.05 from T1, T2, T3, and T4. No differences were observed in between treatments. Data presented are means ± SD

Blood lactate data are shown in Fig. 1. No significant supplement effects were observed [*P* = 0.98]. The lactate responses in S and SCF were similar to CT and P despite a greater volume load and repetition number performed during each protocol. A significant time effect was observed [*P* < 0.0001] where blood lactate was significantly elevated at T3, T4, and T5 compared to T1 and T2 with T3 showing the highest values immediately post resistance exercise.

## Discussion

The unique finding of the present study was that supplementation with 100 mg of *p*-synephrine alone and in combination with 100 mg of caffeine significantly augmented resistance exercise performance compared to P and CT treatments. The SCF and S treatments produced significantly greater numbers of repetitions performed than CT and P and a 10.6 ± 12.0 % greater increase in volume load per protocol than CT and P. Mean power and velocity for all 6 sets were significantly higher in SCF compared to CT and P. No supplement effects were observed in RPE or blood lactate and no side effects were reported throughout the experimental period.

The results of the present study showed ergogenic effects of S and SCF supplementation for increasing repetition performance and volume load during resistance exercise. Both S and SCF treatments led to a nearly 3-repetition augmented performance across all sets although most of the enhancement was seen during the last 3–4 sets. To our knowledge, this is first study to demonstrate an ergogenic potential of *p*-synephrine supplementation when consumed solely or in combination with caffeine during resistance exercise.

The mechanism(s) leading to the enhanced performance remains unknown. The thermogenic actions of *p*-synephrine are thought to be mediated mostly by β-3 adrenoceptor activation predominantly in adipose tissue with weak binding affinity to β-1 and β-2-adrenoceptors [14, 15]. Other studies have shown *p*-synephrine can increase liver glycogenolysis [33] and increase skeletal muscle glucose uptake [34]. The extent to which these lipolytic and metabolic effects of *p*-synephrine augment resistance exercise performance remains unknown and may depend upon how much substrate depletion serves as a performance limiting factor (i.e. for 6 sets of barbell squats in the present study). Recent evidence has shown skeletal muscle possesses β-3 adrenoceptors [35, 36] although specific activation pathways influencing muscle strength, power, and endurance remain speculative. Murphy et al. [35] studied the effects of the β-3 agonist BRL 37344 on rat soleus muscles and reported increased Na+/K+ ATPase pump activity that was mediated through β-2 adrenoceptors. BRL 37344 facilitated intracellular Na+ removal and induced rapid force recovery after rat soleus muscles were fatigued by 16 % following incubation with potassium. Using a more selective β-3 agonist CL 316,243, Miniaci et al. [36] reported increased skeletal muscle protein synthesis via the PI3K-mTOR-p70(S6k) signaling pathway. Taken together, there is growing evidence that β-3 receptor agonists may influence skeletal muscle contractility either directly or indirectly through β-2 adrenoceptor activation. Although the mechanism(s) remains to be elucidated, the effects of *p*-synephrine on skeletal muscle performance via either β-3 adrenoceptor stimulation, possible cell signaling from other β adrenoceptors, or CNS activation requires further study.

Interestingly, the addition of 100 mg of caffeine to 100 mg *p*-synephrine did not augment repetition performance and volume load. Similar findings were reported where the addition of caffeine failed to augment the effects of ephedrine [37, 38]. Caffeine, by itself and in combination with other nutrients, has been shown to be ergogenic for various components of sports performance, aerobic endurance, and anaerobic power [16, 18, 20]. Various studies have shown caffeine enhances resistance exercise performance [18], isometric and isokinetic peak torque and power [19, 21, 23, 39], the number of repetitions performed to failure [24, 40–42] and maximal strength [43], and attenuates reductions in maximal strength and power seen during morning hours [44]. However, some studies have shown caffeine consumption did not augment peak torque [45], 1RM strength [25, 43, 46], or maximal repetition performance/volume load for at least some exercises assessed [38, 43, 46].

Trained subjects may be more responsive to caffeine intake [18, 21, 47] and the likelihood of improvement increases with large muscle group size and location [18, 23, 27, 42, 48]. In a meta-analysis, Warren et al. [27] reported that caffeine exhibited some level of ergogenic improvements in 23 of 27 studies [augmented maximal voluntary contractile (MVC) strength of ~4 % and local muscle endurance by 14 %]. Astorino & Roberson [47] reviewed 11 studies and concluded that caffeine was more likely to increase resistance exercise repetition number (mean improvement of approximately 9.4 %) than 1RM strength.

The vehicle of consumption and dose of caffeine affects the acute response. Most studies demonstrating ergogenic effects of caffeine utilized anhydrous caffeine consumption at moderate-to-high doses typically ranging from 2 to 9 mg/kg of body mass [27, 47]. Astorino et al. [49] reported only a higher dose of caffeine (5 versus 2 mg/kg) improved isokinetic resistance exercise performance by 5–8 % thereby demonstrating a dose–response relationship. In the present study caffeine intake was low and given in a standard absolute amount within each chew averaging 1.23 mg/kg of body mass. Thus, it is possible that a higher dose of caffeine is needed to augment repetition performance and volume load. However, the potentiating effect of *p*-synephrine consumed in combination with caffeine needs to be considered. *p*-Synephrine alone augmented resistance exercise performance similarly to the SCF treatment. The amount of *p*-synephrine consumed in the present study was higher than most studies [11] but similar to one study [13] although these studies did not examine resistance exercise performance. Our data indicate that the addition of only 100 mg of caffeine to the chews may not be sufficient to augment further increases in local muscle endurance.

The addition of caffeine to *p-*synephrine did augment repetition average velocity and power. Average power and velocity for all 6 sets were significantly higher in SCF compared to CT and P by ~6.2 ± 8.0 %. Although mean power during S was higher than CT and P (by ~3 %), these values did not reach statistical significance. The mechanisms of caffeine’s ergogenic effects are vast and difficult to isolate during exercise. Caffeine is a central nervous system stimulant that increases lipolysis, potentiates cyclic AMP via phosphodiesterase inhibition, and augments post-exercise muscle glycogen storage [20]. However, during high-intensity resistance exercise other mechanisms appear more plausible. Caffeine may increase contractile force through mobilization of intracellular calcium, increased calcium release from the sarcoplasmic reticulum, and decreased calcium uptake [20]. Other resistance exercise studies have shown increased muscle fiber conduction velocity [19] and EMG activity [39] following caffeine consumption possibly indicative of greater motor unit recruitment [27]. Adenosine antagonism, reduced pain perception, and blunted perceived exertion during high-intensity exercise performance may also play pivotal roles [18, 24, 40]. Thus, it appears multiple mechanisms in combination could contribute to caffeine’s ergogenic effects of average velocity and power observed during each set.

An interesting finding was that the addition of 100 mg of caffeine to 100 mg of *p-*synephrine augmented average power during each set despite similar total repetitions and volume load performed between S and SCF treatments. Further analysis showed that the average and peak power obtained during each repetition that preceded muscular failure was still greater in SCF. This indicates that the subjects performed more powerful repetitions up until the point of momentary muscular exhaustion. However, the enhanced power did not result in a greater number of repetitions or volume load performed in SCF compared to S. In fact, no significant correlations were shown between average or peak power values and the number of repetitions completed. Similar findings were reported by Behrens et al. [50] who showed that caffeine consumption increased power performance of the plantar flexor muscles but did not increase MVC strength. One plausible reason may be that maximal and near-maximal squat performance is limited by the sticking region (area where bar velocity is minimal) located superior to the bottom parallel position close to an absolute thigh angle of approximately 30° [51]. Surpassing the sticking region increases the likelihood of completing a successful repetition as bar velocity increases during the remainder of the range of motion until the deceleration phase ensues [51]. Although power and velocity were higher during each repetition, it is possible the greater power did not transfer through the sticking region when fatigue reached maximal levels. Nevertheless, the higher power and velocity values observed during the SCF treatment may be viewed as beneficial and could potentially lead to greater power gains during long-term training periods.

Average and peak concentric force decreased from set 1 through set 6 at various points. However, no supplement effect was observed with regard to the pattern of reduction during each set. Although S and SCF treatments yielded higher peak concentric force values (of up to 2.5 %) than CT and P, respectively, these differences did not reach statistical significance. These data demonstrate that the velocity increase was the main contributor to increased power seen in SCF. Although force and velocity are positively related, the small relative peak concentric force increases observed in SCF and S did not reach statistical significance. Nevertheless, this small mean difference appeared large enough to increase bar velocity and subsequent power performance.

The results of the present study showed a progressive increase in RPE during each successive set with no supplement effects observed. Numerous studies have examined the effects of caffeine on RPE. These studies have shown either no effect of caffeine on RPE [41–43, 46, 49] or a reduced RPE response [24, 40] during resistance exercise. Doherty and Smith [52] conducted a meta-analysis of studies examining caffeine effects on RPE during exercise and concluded that caffeine reduced RPE by 5.6 % with a concomitant 11.2 % increase in performance. RPE accounted for 29 % of the variance in exercise performance [52]. Haller et al. [11] examined Ripped Fuel (21 mg of synephrine and 304 mg of caffeine) during 30 min of cycling at 75–80 % VO_2_ max and reported lower RPE during exercise. Thus, our data support studies during resistance exercise showing similar RPE values between caffeine and placebo conditions. The results of the present study indicated that *p*-synephrine alone or in combination with caffeine yielded similar RPE during resistance exercise. Considering that significant performance improvements were seen in both S and SCF treatments, these data indicate that both supplements can blunt acute increases in RPE that would be expected with a greater volume load of resistance exercise.

A similar finding was observed in blood lactate. Significant elevations in blood lactate were seen at T3, T4, and T5 with no differences observed between treatments. Haller et al. [11] reported no difference in blood lactate response between Ripped Fuel and placebo treatments during cycling. Studies examining the effects of caffeine consumption on blood lactate response to resistance exercise have shown no differences [40] or greater blood lactate concentrations [24] as performance enhancement was shown. The results of the present study indicate that 100 mg of *p*-synephrine increased repetition performance and volume load without increasing the blood lactate response. The addition of 100 mg of caffeine did not augment the response. Thus, both the S and SCF supplements enhanced performance without eliciting a greater lactate response.

The results of the present study should be viewed within the design limitations. The supplementation protocol was based on using absolute dosing in lieu of relative dosing as the intent was to examine the supplements in their available chew form. In addition, subjects consumed the supplements for three days prior to performing each protocol. Thus, the acute resistance exercise response to a single dose remains to be seen. Future studies should address *p*-synephrine supplementation using a variety of doses for different types of resistance exercise programs and should examine potential chronic training effects.

## Conclusions

This study demonstrated that 100 mg of *p-*synephrine alone or in combination with 100 mg of caffeine significantly augmented resistance exercise repetition performance and volume load. The addition of caffeine to *p*-synephrine resulted in faster, more powerful repetitions. These changes took place without a concomitant increase in RPE or blood lactate indicating that *p*-synephrine (with and without caffeine) supplementation can blunt acute increases in RPE and blood lactate known to accompany a greater volume of exercise. The resultant effects are increased local muscular endurance and more powerful repetitions (when caffeine is added) with no additional perceived exertion or lactate accumulation.
